# Physiologic heart rate dependency of the PQ interval and its sex differences

**DOI:** 10.1038/s41598-020-59480-8

**Published:** 2020-02-13

**Authors:** Ondřej Toman, Katerina Hnatkova, Peter Smetana, Katharina M. Huster, Martina Šišáková, Petra Barthel, Tomáš Novotný, Georg Schmidt, Marek Malik

**Affiliations:** 1 Department of Internal Medicine and Cardiology, University Hospital Brno, Faculty of Medicine, Masaryk University, Jihlavská 20, 625 00 Brno, Czech Republic; 2 National Heart and Lung Institute, Imperial College, 72 Du Cane Rd, Shepherd’s Bush, London, W12 0NN England; 3 Wilhelminenspital der Stadt Wien, Montleartstraße 37, 1160 Vienna, Austria; 4 Klinikum rechts der Isar, Technische Universität München, Ismaninger Straße 22, D-81675 Munich, Germany

**Keywords:** Cardiology, Medical research

## Abstract

On standard electrocardiogram (ECG) PQ interval is known to be moderately heart rate dependent, but no physiologic details of this dependency have been established. At the same time, PQ dynamics is a clear candidate for non-invasive assessment of atrial abnormalities including the risk of atrial fibrillation. We studied PQ heart rate dependency in 599 healthy subjects (aged 33.5 ± 9.3 years, 288 females) in whom drug-free day-time 12-lead ECG Holters were available. Of these, 752,517 ECG samples were selected (1256 ± 244 per subject) to measure PQ and QT intervals and P wave durations. For each measured ECG sample, 5-minute history of preceding cardiac cycles was also obtained. Although less rate dependent than the QT intervals (36 ± 19% of linear slopes), PQ intervals were found to be dependent on underlying cycle length in a highly curvilinear fashion with the dependency significantly more curved in females compared to males. The PQ interval also responded to the heart rate changes with a delay which was highly sex dependent (95% adaptation in females and males after 114.9 ± 81.1 vs 65.4 ± 64.3 seconds, respectively, p < 0.00001). P wave duration was even less rate dependent than the PQ interval (9 ± 10% of linear QT/RR slopes). Rate corrected P wave duration was marginally but significantly shorter in females than in males (106.8 ± 8.4 vs 110.2 ± 7.9 ms, p < 0.00001). In addition to establishing physiologic standards, the study suggests that the curvatures and adaptation delay of the PQ/cycle-length dependency should be included in future non-invasive studies of atrial depolarizations.

## Introduction

The electrocardiographic (ECG) interval between the onset of the P wave and the onset of the QRS complex is frequently called the PR interval but should be more accurately termed the PQ interval. As well known, the duration of this interval represents the combination of the intra-atrial conduction and of the excitation delay within the atrioventricular node.

Abnormalities of the PQ interval are associated not only with the atrioventricular node pathologies but also with intra-atrial anomalies. In addition to the diagnosis of atrioventricular conduction pathologies, monitoring of the PQ interval is therefore useful for the assessment and device programming of cardiac resynchronization therapy^1–3^ as well as for clinical follow-up of heart failure patients^4,5^. Prolongation of the PQ interval was also found predicting adverse outcome in general population^6,7^. More recently, a number of studies showed that PQ interval may be used as a risk indicator of subsequent development of atrial fibrillation or of its recurrence after ablation therapy^8–11^. Similarly, risk of atrial fibrillation was reported to relate to the P wave duration^12,13^.

Similar to the ventricular conduction system, intra-atrial conduction is influenced by the autonomic nervous system^14^. It is therefore not surprising that PQ interval duration also exhibits autonomically modulated changes and that the interval is heart rate dependent^15,16^. Also not surprisingly, some of the characteristics of autonomic PQ adaptation have been reported to predict atrial fibrillation^17^. Nevertheless, details of the heart rate dependency of PQ interval are sparse^16,18^. Although it might be expected that more accurate characterization of the dependency in individual subjects would further advance the clinical utility of the PQ monitoring, little has been published on the autonomic modulations of the P wave duration.

Previous studies only examined simple linear relationships between the PQ interval durations and either underlying heart rate or the simultaneously measured RR intervals. Physiologic speed of the adaptation of the PQ interval in response to heart rate changes and non-linear patterns of the relationship have not been researched. Likewise, very limited data exist on the possible sex and age differences in the PQ/heart rate relationship.

This limited understanding of the character of PQ dynamics prevents the development of more accurate non-invasive methods for the assessment of intra-atrial conduction abnormalities including the risk of atrial fibrillation. Therefore, to address these knowledge gaps, we used large sets of ECG data obtained in healthy subjects both to develop and test methodology of the intra-subject characterization of the PQ/heart rate relationship, and to statistically summarize the results in terms of the age dependency and of the differences between healthy females and males. Since substantial experience exists with the dynamics of the QT interval and with intra-subject assessment of the QT/heart rate relationship, we also compared the PQ interval dependency on heart rate with that of the QT interval assessed in the very same population.

## Methods

### Investigated population and electrocardiographic recordings

Clinical pharmacology studies were conducted at 6 different clinical research units and enrolled altogether 639 healthy subjects. All participating subject were screened before enrolment and all had a normal resting ECG and normal clinical investigation as mandated prior to clinical pharmacology research studies^19^. All these source studies were ethically approved by the institutional ethics bodies (Focus in Neuss; Parexel in Baltimore, Bloemfontein, and Glendale; PPD in Austin; and Spaulding in Milwaukee) and all subjects gave informed written consent to the participation in the study in accordance with the Helsinki declaration.

Age and demographic data were obtained in the subjects at the time of enrolment to the source studies. Body mass index was calculated as body weight in kilograms divided by the square of body height in metres. The subjects were drawn from standard pool of volunteers of clinical pharmacology studies and did not include highly trained athletes.

Each of the studies included repeated 12-lead day-time ECG Holter recordings in each participant. The recordings were made during multiple baseline days when the subjects were on no treatment. During these baseline days, study protocols included repeated provocative manoeuvres with the aim of capturing wide heart rates ranges on the recordings of each participant. The Holter recordings used Mason-Likar electrode positions.

The investigation described in this text utilized the baseline Holter recordings when the subjects were off any medication, did not smoke, and refrained from consuming caffeinated drinks. Also, the baseline handling of the study participants did not differ between individual clinical research units. Consequently, further details of the clinical pharmacology studies are of no relevance.

### Electrocardiographic measurements

Using previously developed technology combining computerized signal processing with visual checks and manual corrections of the measurements^20^, P wave onset, QRS onset, and T wave offset points were identified in multiple sections of each of the 12-lead Holter recordings. (In addition to these measurements, QRS offset points were also identified but these were not used in the data analyses reported here.) The measurements were made in representative median beats, sampled at 1000 Hz, derived from 10-second ECG segments of the Holter recordings. Pattern matching algorithms^21^ were included in the measurement set-up. This ensured that comparable morphologies of P wave onset, QRS onset, and T wave offset were measured in a corresponding way. Quality control of the measurements was also used. This included visual verification and manual correction of computerized measurements by at least two independently working cardiologists with subsequent independent reconciliation in case of measurement disagreement. The visual inspection by the two independent cardiologists also included a decision of whether a morphological point can be defined with sufficient certainty. Additionally, P wave offsets were identified at the decrease of the root mean square of 12-lead P wave voltages below 15% of its maximum value.

For each 10-second ECG segment in which the morphological point identifications were made, a 5-minute history of RR interval preceding the segment was also obtained. This allowed to include measurements preceded by both stable and variable heart rates during the preceding 5 minutes.

In each measured ECG segment, PQ interval was defined as the time distance between the P wave onset and the QRS onset, while the QT interval was defined between the QRS onset and the T wave offset. P duration was the distance from P wave onset and offset.

### Heart rate and rate hysteresis correction

#### Relationship to heart rate

Since multiple ECG measurements over broad ranges of underlying heart rates were available in each subject, we had the possibility to investigate intra-subject relationship of PQ intervals (and similarly QT intervals and P wave durations) to heart rate. For this purpose, 4 different regression models were considered. In the following descriptions of these models, symbols ${{\rm{PQ}}}_{i}$ represent repeated measurements of the PQ interval in the same subject, ${{\rm{RR}}}_{i}$ are the RR interval durations corresponding to the underlying heart rate of the ${{\rm{PQ}}}_{i}$ measurements, ${\varepsilon }_{i}$ are zero centred normally distributed residual errors, and ${\rm{PQc}}$ is the regression projected value of the PQ interval at the heart rate of 60 beats per minute, i.e. at RR = 1 s. The values of ${{\rm{PQ}}}_{i}$ and of ${{\rm{RR}}}_{i}$ were considered in seconds.

- *Linear model* assumed that PQ intervals were linearly related to the underlying RR intervals and was based on the regression ${{\rm{PQ}}}_{i}={\rm{PQc}}+\alpha ({{\rm{RR}}}_{i}-1)+{\varepsilon }_{i}$. For each study subject, a subject-specific value of the coefficient $\alpha$ was optimized to achieve zero centred distribution of residual errors ${\varepsilon }_{i}$.
- *Log-linear model* assumed that the logarithms of PQ intervals and of RR intervals were linearly related and was based on the regression $\log ({{\rm{PQ}}}_{i})=\,\log ({\rm{PQc}})+\beta \,\log ({{\rm{RR}}}_{{\rm{i}}})+{\varepsilon }_{i}$. As with the linear model, the subject-specific coefficient $\beta$ was optimized for each study subject.
- *Hyperbolic model* assumed that PQ intervals were linearly related to the underlying heart rate (rather than to the corresponding RR intervals) and was based on the regression ${{\rm{PQ}}}_{i}={\rm{PQc}}+\vartheta (1-1/{{\rm{RR}}}_{i})+{\varepsilon }_{i}$. Again, the coefficients $\vartheta$ were optimized for each subject separately.
- *Curvilinear model* was based on previous experience based on the investigations of the QT/RR relationship^22^ that showed that not only the slopes of the relationship but also the patterns and curvatures of the relationship differ between different healthy subjects. The model was based on the regression ${{\rm{PQ}}}_{i}=\alpha +\frac{\delta }{\gamma }({{\rm{RR}}}_{i}^{\gamma }-1)+{\varepsilon }_{i}$ where the coefficients $\delta$ (the slope of the relationship) and $\gamma$ (the curvature of the relationship) were optimized in each study subject to achieve not only zero centred distribution of the residual errors ${\varepsilon }_{i}$ but also the lowest standard deviation of the residual errors ${\varepsilon }_{i}$. That is, the curvature coefficient $\gamma$ was selected such that the model fitted the PQ/RR data in hand better than with any other value of the curvature coefficient. As previously explained^22^, curvature coefficients $\gamma$ close to 1 indicate linear relationship. The more $\gamma$ differs from 1, the more curved the relationship ($\gamma$ values above and below 1 indicate convex and concave patterns, respectively).

#### Heart rate hysteresis

The concept of heart rate hysteresis (known from the studies of QT interval dynamics^23,24^) relates to the observation that after an abrupt heart rate change, the heart rate dependent ECG characteristic (the PQ interval in this study) may not change immediately but with a certain delay. This speed of the response to heart rate change (i.e. how quickly the characteristic changes) might be independent of the extent of the change (i.e. how much the characteristic changes).

The experience with the heart rate hysteresis of the QT interval duration shows that if a rate dependent characteristic shows rate hysteresis, its relation to instantaneously measured heart rate is less compact compared to the relation to averaged rate over a longer heart rate history^25^. Therefore, to investigate this phenomenon, three different expressions of RR interval value corresponding to heart rate underlying a PQ interval measurement were considered:

- *Average of preceding 3 RR intervals* was used to represent the instantaneous heart rate simultaneous with the PQ interval measurement. Since the measurements were made on representative complexes of ECG segments, the average of last 3 RR intervals of the segment were used including a verification that the PQ intervals in the corresponding heart beats did not differ from the representative complex measurements.
- *Average of 10-second RR intervals* was obtained from the 10-second ECG segment in which the measurement of the PQ interval was made.
- *Hysteresis corrected RR intervals* were obtained using subject-specific optimization of the exponential decay model of heart rate influence^23^. That is, for a PQ interval measurement, the sequence of preceding RR interval ${\{R{R}_{i}\}}_{i=0}^{N}$ ($R{R}_{0}$ closest to the PQ measurement) was considered. The RR interval representing the heart rate underlying the PQ measurement was then calculated as $R{R}^{\text{'}}=\mathop{\sum }\limits_{i=0}^{N}{\omega }_{i}R{R}_{i}$, where for each $j=0,\cdots ,N$,

$$\mathop{\sum }\limits_{i=0}^{j}{\omega }_{i}=\frac{1-{{\rm{e}}}^{-\lambda (\frac{{\sum }_{i=0}^{j}R{R}_{i}}{{\sum }_{i=0}^{N}R{R}_{i}})}}{1-{{\rm{e}}}^{-\lambda }}$$

The coefficient $\lambda$ characterized the subject-specific PQ/RR hysteresis, i.e. the speed with which PQ interval adapted to changing heart rate. As described in the subsequent section, the coefficient $\lambda$ was obtained for each study subject in conjunction with the PQ/RR regression model. The value of $\lambda$ was subsequently converted into the so-called hysteresis time-constant, i.e. the time needed for the PQ interval to reach 95% of its new value after a heart rate change.

#### Investigations of the relationship to heart rate

To investigate the character of the PQ heart rate dependency, we used the following prospectively designed investigation plan of two steps:

The first step evaluated the consistence of PQ/RR hysteresis. Specifically, to check whether PQ/RR hysteresis needs to be considered, the linear, log-linear, and hyperbolic models were combined with all three RR interval expressions. In case of the hysteresis corrected RR intervals, the coefficient $\lambda$ was always optimized (in each study subject separately) to achieve the lowest standard deviation of the residual errors ${\varepsilon }_{i}$ (i.e. to obtain the closest fit of the chosen regression model).

Since the residual errors ${\varepsilon }_{i}$ were always zero centred, their standard deviation was a valid characterization of the compactness of the regression fit - it is an estimate of the sum of squares of the differences between the regression line or curve and the actual PQ interval measurements at the corresponding heart rate. We call these standard deviations the *regression residuals*.

The hysteresis-type adaptation of the PQ interval to changing heart rate was established if, in the study population, the regression residuals of the PQ interval regressions to hysteresis corrected RR intervals were smaller than the residuals of the PQ regressions to other RR interval expressions. In that case, the hysteresis time constants were considered in the description of the PQ/RR relationship.

The second step evaluated the curvature of PQ/RR patterns. Using the RR interval expression determined in the first step, a comparison was made between the different PQ/RR regression models. Subject-specific curvatures of the PQ/RR relationship were established if, in the study population, the regression residuals of the curvilinear PQ/RR regression model were smaller compared to the regression residuals of the other PQ/RR regression models. For the combination of the curvilinear PQ/RR model with the hysteresis corrected RR intervals, both parameters $\lambda$ (hysteresis profile) and $\gamma$ (curvature of the PQ/RR relationship) were optimized in each subject of the study by searching for the minimal regression residual within the complete 2-dimensional $[\lambda |\gamma ]$ space.

#### Minimising regression residuals

The validity of characterisation of the heart rate relationship of an ECG interval is dependent on finding an appropriate model describing the relationship. While the slopes of linear regressions approximate the overall extent of the heart rate dependency (e.g. may be used to estimate whether an ECG interval is more dependent on heart rate than another ECG interval) it is obvious that linear models cannot accurately describe relationships that show highly curved patterns. This is the reason why we used curvilinear models and employed the regression residuals as a guide for the selection of the most appropriate model.

Minimizing the regression residuals allows to find the optimum PQ/RR relationship that finds how much of the PQ (or QT) variability may be explained by the heart rate dependency. In other words, the regression residuals measure the goodness of the fit of the regression through the cloud of the PQ and RR data in each study subject. This allows to compare different regressions by statistical comparisons of their regression residuals across the study population.

This also allows selecting the optimum regression model for each study subject separately which in turn allows to compare not only the slopes of the rate-relationship but also its curvatures and the hysteresis time constants between different ECG intervals as well as between different populations sub-groups (e.g. females and males as reported in this study).

#### Heart rate correction

In each subject, the optimum model of the PQ/RR relationship (i.e. the combination of the PQ/RR regression with the RR interval expression selected by procedure described in the previous section) was used with the subject-specific coefficients. The projection of this regression to the value of RR = 1 s was used as the individually heart rate corrected duration of the PQ interval. This value is termed the PQc interval.

#### Comparison between PQ, P wave and QT interval heart rate relationship

The very same methodology of combining the four different regression models with the three RR interval expressions was also applied to the QT/RR and P-wave-duration/RR relationships in the study subjects (including the calculation of the individually corrected QTc intervals and rate corrected P wave durations). This allowed us to study paired comparisons between the subject-specific parameters of the PQ/RR, P-duration/RR, and QT/RR relationship.

### Summaries of data in heart rate bins

To validate the results obtained with the curvilinear regression models, we used the so-called heart rate bin analysis^26,27^ that allows studying the rate relationship of the investigated ECG intervals without involving any heart rate correction and/or regression modelling.

For each study subject and each heart rate level, all ECGs of the given subject were selected for which the underlying heart rate (with appropriate hysteresis correction) did not differ from the given heart rate level by more than a pre-specified width of a sampling bin. The medians of the ECG interval measurements (of PQ and QT intervals and of P wave durations) characterized the durations of the measured intervals in the given subject at the given heart rate. In each heart rate bin, these median values of separate subjects were subsequently statistically summarized (separately for females and males) by calculating their means, standard deviations, and 99% confidence intervals derived from normal distribution.

### Statistics and data presentation

Continuous data are presented as means ± standard deviation or as medians and inter-quartile ranges, as appropriate. The significances of intra-subject paired differences of regression residuals or of the differences between corresponding characteristics of the PQ/RR and QT/RR relationships were tested using paired two-tail t-test. The differences between female and male sub-populations were tested by two-sample two-tail t-test assuming unequal variances of the compared distributions. Age dependencies of the characteristics were investigated by Pearson correlation coefficients and, where appropriate, displayed as linear regressions shown together with their 95% confidence bands. The statistical tests were calculated using the IBM SPSS Statistics package, version 25. P-values below 0.05 were considered statistically significant. When repeating statistical tests for 6 sub-populations per source clinical centres, Bonferroni correction of the statistical significance was used; no other corrections were considered.

## Results

### Population and electrocardiographic measurements

The source clinical pharmacology studies investigated 639 subjects (311 females). Of these, 40 (6.26%, 23 females) had to be subsequently excluded since the optimization algorithms for the calculation of PQ/RR hysteresis profile did not converge. The population of the reported investigation therefore comprised 599 subjects (288 females aged 33.5 ± 10.1 years, and 311 males aged 33.6 ± 8.5 years, no statistical difference between the ages of the sex sub-groups). In the baseline Holter recordings of these subjects, ECG measurements were attempted in 752,517 samples. Of these, PQ and QT intervals and P wave durations were measurable in 747,651 (99.4%), 749,144 (99.6%) and 726,181 (96.5%) respectively. There were no significant differences between the numbers of PQ measurements in individual females (1,237 ± 259) and males (1,259 ± 237) or between the numbers of P wave duration measurements in individual females (1,210 ± 260) and males (1,223 ± 250). Table 1 show the distribution of the study population in the 6 clinical centres.

**Table 1:** Subject and data distribution in separate clinical centres.

Centre	Females					Males				
	N	Age [years]	PQ data	P data	QT data	N	Age [years]	PQ data	P data	QT data
1	83	32.7 ± 9.4	1385	1360	1383	93	31.9 ± 7.7	1421	1397	1423
2	81	32.1 ± 10.2	1310	1279	1306	75	35.3 ± 8.4	1345	1290	1348
3	13	22.9 ± 2.0	1311	1274	1315	15	24.6 ± 4.6	1192	1176	1192
4	16	52.3 ± 6.0	1447	1415	1447	15	48.5 ± 7.6	1553	1487	1556
5	72	34.6 ± 6.9	1031	1004	1034	90	34.1 ± 6.6	1036	999	1049
6	23	30.3 ± 9.8	900	895	902	23	29.4 ± 7.6	1043	1012	1046
ALL	288	33.5 ± 10.1	1237	1210	1236	311	33.6 ± 8.5	1259	1223	1264

For each clinical centre, the table gives the number (N) of female and male subjects, their average (±standard deviation) ages, and averaged numbers of ECG segments with valid PQ intervals, P wave duration, and QT interval data per subject. The line ALL shows the same data for all centres combined. In all clinical centres (and in the combined data of all centres), there were no statistically significant differences between the ages of the female and male subjects.

**Figure 1:**
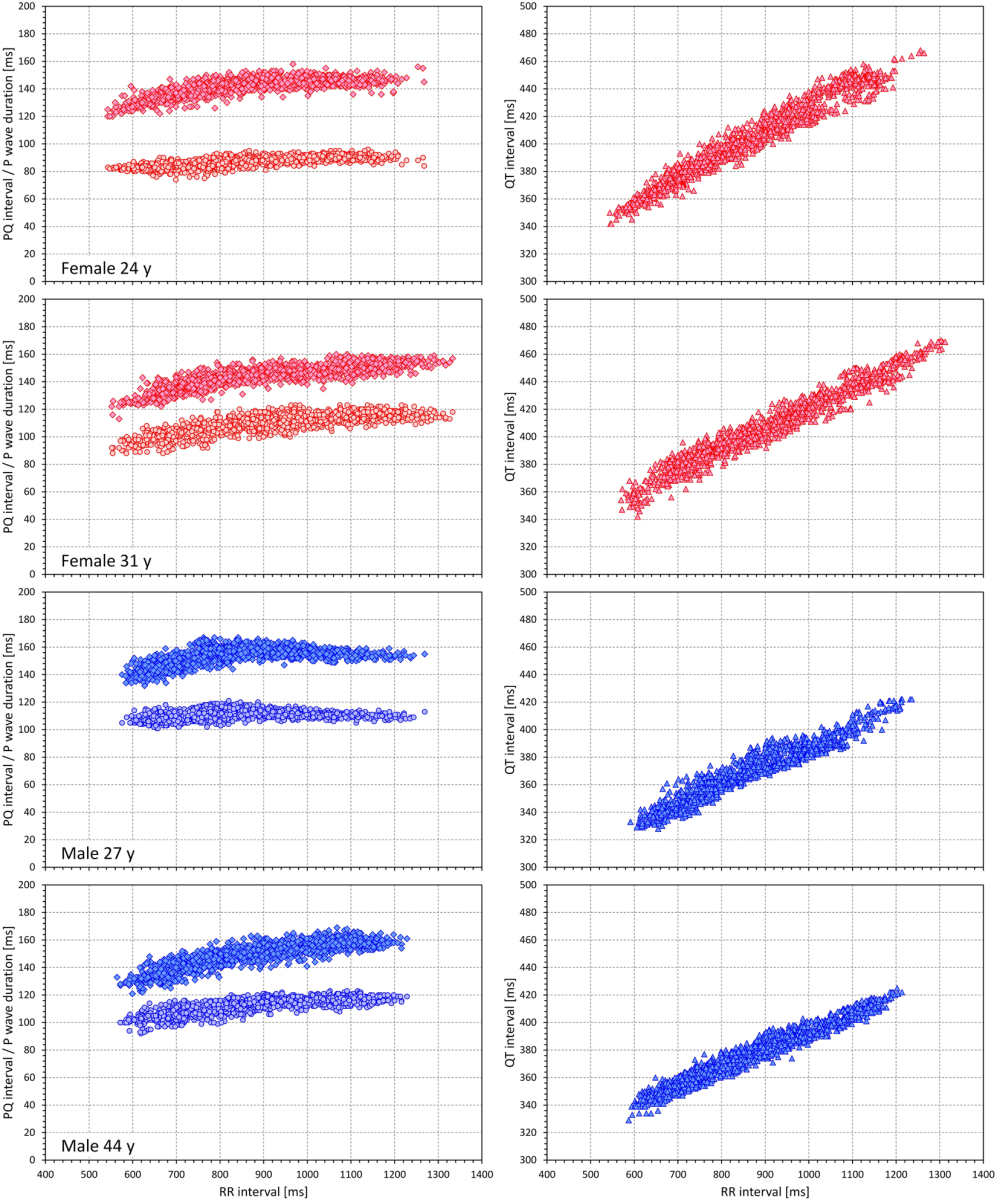
Examples of individual PQ/RR relationship in comparison to the QT/RR relationship in the same individual. Each row of two panels corresponds to one subject (from top to bottom: females aged 24 and 31 years, males aged 27 and 44 years – the diagrams of females and males are in red and blue, respectively). The panels on the left show the PQ/RR relationships (diamonds) and P-wave-duration/RR relationship (circles), the panels on the right show the QT/RR relationships. In each panel, the original PQ and QT measurements are shown against the hysteresis corrected RR intervals after subject-specific hysteresis optimization (see the text for details). Note that while the panels on the left and on the right have different vertical axes, the span of the axes is always 200 ms allowing the comparison between the steepness of the PQ/RR and QT/RR relationships.

PQ/RR and P-wave-duration/RR patterns were first judged visually. Figure 1 shows examples of intra-subject PQ/RR relationships in 4 study subjects and allows the comparison with the QT/RR relationships in the same individuals. The figure is in agreement with previous reports^16,18^ that showed much lower slopes of PQ/RR profiles compared to QT/RR profiles. The figure also suggests that the PQ/RR profiles are more curved. The figure further shows that while the P wave durations are not stable over different heart rates, the rate dependency of P wave durations is even shallower compared to that of the PQ intervals.

### Heart rate hysteresis assessment

Table 2 shows the comparison of regression residuals of the combinations of the linear, log-linear and hyperbolic regression models with different RR interval expressions. For all these regression models, the PQ/RR regression residuals obtained with the hysteresis corrected RR intervals were highly significantly smaller compared to the other RR interval expressions (p < 0.00001 in all cases). Consistent with previous reports^23^, the same observation was also made with the QT/RR regression residuals for which the differences between the RR interval expressions were even larger (again, p < 0.00001 in all cases).

**Table 2:** Regression residuals.

Regression model	Females			Males		
	3 intervals	10 seconds	hysteresis	3 intervals	10 seconds	hysteresis
**PQ/RR relationship**						
linear	8.11 ± 2.45	7.97 ± 2.43	7.33 ± 2.25	7.42 ± 1.97	7.18 ± 1.95	6.68 ± 1.83
log-linear	8.56 ± 2.68	8.43 ± 2.66	7.79 ± 2.46	7.69 ± 2.10	7.42 ± 2.06	6.92 ± 1.94
hyperbolic	7.94 ± 2.39	7.78 ± 2.38	7.11 ± 2.19	7.25 ± 1.90	6.95 ± 1.88	6.47 ± 1.77
curvilinear			6.96 ± 2.12			6.33 ± 1.73
**QT/RR relationship**						
linear	11.83 ± 2.28	10.73 ± 2.03	5.91 ± 1.18	11.46 ± 2.08	10.57 ± 1.95	5.58 ± 1.09
log-linear	12.67 ± 2.39	11.54 ± 2.15	6.31 ± 1.23	12.09 ± 2.12	11.16 ± 1.95	5.86 ± 1.14
hyperbolic	12.17 ± 2.30	11.07 ± 2.10	6.61 ± 1.43	11.97 ± 2.18	11.04 ± 2.02	6.48 ± 1.42
curvilinear			5.68 ± 1.12			5.43 ± 1.07

For linear, log-linear and hyperbolic regression models (see the text for details) the table shows regression residuals (mean ± standard deviation, values in milliseconds) for RR interval expressions as the average of 3 RR intervals (3 intervals), the average of 10 seconds of RR intervals (10 seconds) and the individually hysteresis corrected RR intervals (hysteresis). Regression residuals are also shown for the combination of curvilinear regression models with the hysteresis corrected RR intervals. Results of the PQ/RR and QT/RR relationship are shown in the top and bottom part of the table, respectively. The results in females and males are shown in the left and right parts of the table, respectively.

Interestingly, while on average, the linear QT/RR regression model fitted the intra-subject profiles better than the log-linear or hyperbolic models, the hyperbolic PQ/RR model provided lower residuals compared to the linear and log-linear models (again, p < 0.00001 for all comparisons). This is in agreement with the visual observations of substantially curved PQ/RR patterns seen in Fig. 1.

**Figure 2:**
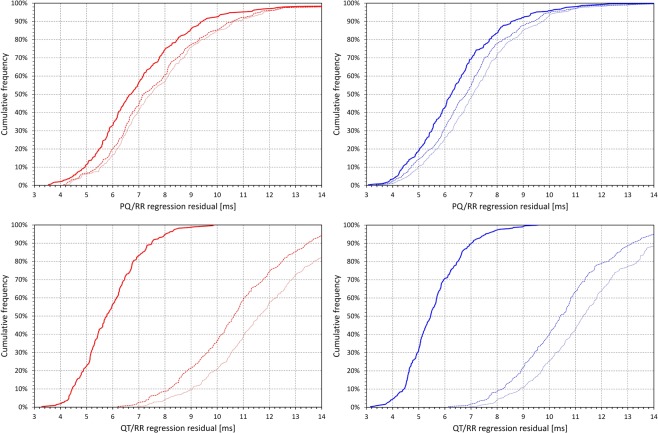
Cumulative distributions of the regression residuals (see the text for details) of the hyperbolic PQ/RR regression models in the top row and of the linear QT/RR regression models in the bottom row. The panels on the left with red lines show the distribution in females, the panels on the right with blue lines the distribution in males. The dotted, dashed, and bold lines show the distribution of the residuals obtained with the averages of 3 RR intervals, 10-second averages of RR intervals, and hysteresis corrected RR intervals, respectively.

Figure 2 shows the cumulative distributions of the regression residuals obtained with different RR interval expressions. Because of the comparison with other models, hyperbolic PQ/RR and linear QT/RR models are shown. The figure demonstrates that incorporating hysteresis correction of the RR intervals led to substantially larger residual reduction of the QT/RR models compared to the PQ/RR models. Nevertheless, while the PQ/RR regression residuals overlap in the complete population, the hyperbolic PQ/RR regression residuals involving hysteresis corrected RR intervals were larger than the hyperbolic PQ/RR regression residuals based on either of the other RR interval expressions only in 1 female (0.35%) and in 3 males (0.96%).

Hence, this analysis proved that PQ interval duration does not adapt to the heart rate changes instantaneously but with a delay and that the heart rate phenomenon of heart rate hysteresis needs to be considered.

### Curvature of heart rate dependency

Table 2 also shows the regression residuals of the curvilinear PQ/RR and QT/RR regression models combined with hysteresis corrected RR intervals. Similar to the QT/RR regressions, the residuals of the curvilinear PQ/RR regressions were significantly smaller compared to the residuals of the other regression models (p < 0.00001 for all comparisons). Using the combination with hysteresis corrected RR intervals (i.e. with subject-specific hysteresis corrections), the curvilinear model had lower intra-subject regression residuals than the linear and hyperbolic models for every study subject. The log-linear PQ/RR model had lower regression residuals than the curvilinear model only in 4 males (1.28%) and in no females.

**Figure 3:**
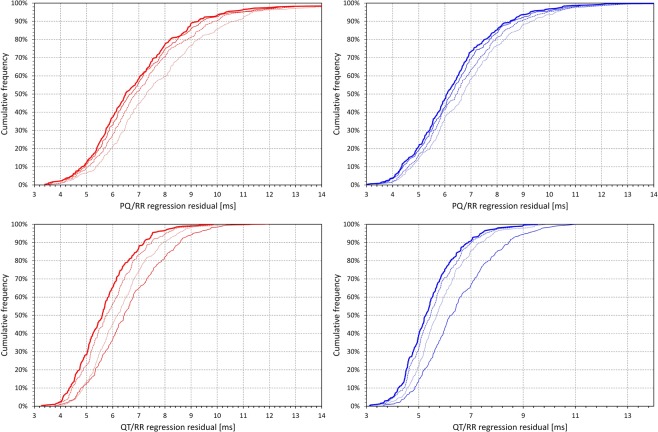
Cumulative distributions of the regression residuals (see the text for details) of the PQ/RR regression models in the top row and of the QT/RR regression models in the bottom row. The panels on the left with red lines show the distribution in females, the panels on the right with blue lines the distribution in males. In all cases, the regressions involved hysteresis corrected RR intervals. The fine dotted lines, fine dashed lines, fine bold lines, and bold full lines correspond to log-linear, linear, hyperbolic, and curvilinear regression models, respectively.

The cumulative distributions of the regression residuals of different regression models combined with the subject-specific hysteresis corrections are shown in Fig. 3. Albeit somewhat smaller, the reduction in PQ/RR regression residuals when involving the curvilinear model is reminiscent of the corresponding QT/RR residual changes.

The difference in the P-wave-duration/RR residuals was smaller still, albeit statistically significant. In females, the curvilinear and linear P-wave-duration/RR models led to residuals of 5.15 ± 1.52 and 5.26 ± 1.53 ms, respectively (p < 0.0001) while in males, the corresponding numbers were 5.24 ± 1.85 and 5.37 ± 1.85 ms, respectively (p < 0.0001). Hence, the curvatures of the P-wave-duration/RR relationship were also statistically confirmed but the comparisons are not shown in Fig. 3 because of the miniscule numerical differences.

### Heart rate dependency characteristics

#### Hysteresis time constants

**Figure 4:**
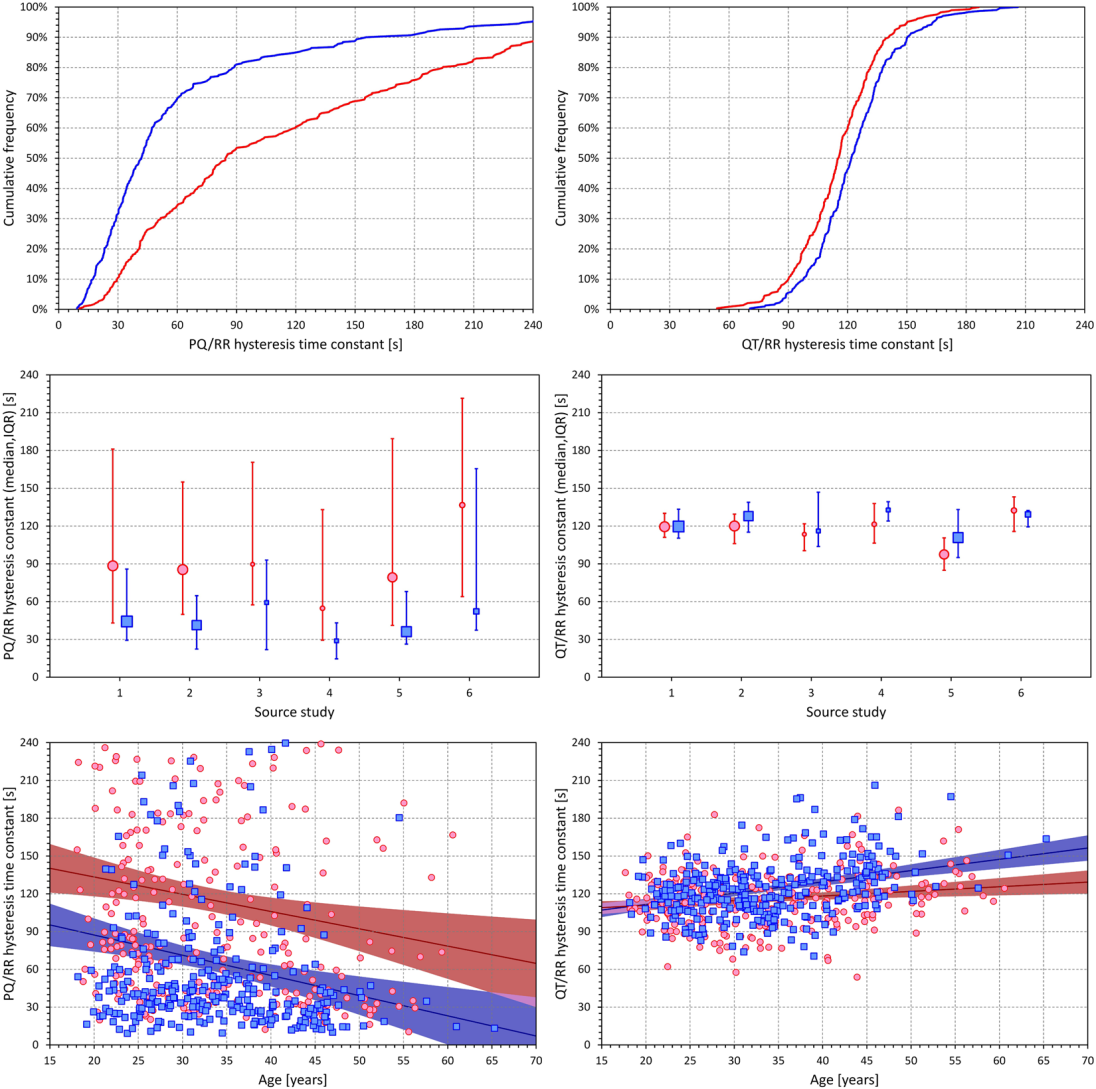
The panels on the top row show the cumulative distribution of subject-specific hysteresis constants of the curvilinear regression models of the PQ/RR relationship on the left and of the QT/RR relationship on the right. The curves of the red and blue lines correspond to the distributions in females and males, respectively. The panels of the middle row show comparisons of the hysteresis time constants between females (red circles with red error bars) and males (blue squares with blue error bars) in sub-populations defined by the source clinical centre of the study (see the text for details). The graphs show medians and inter-quartile ranges, the sizes of the marks indicate the numbers of subjects in the sub-populations. The panels on the bottom row show the scatter diagrams of the individually optimized hysteresis time constants against the ages of the study subjects. The red/pink circles correspond to females, the blue/azure squares to males. In each panel, the solid red and solid blue lines show the linear regressions between the hysteresis time constants and age in females and males, respectively. The red shaded and blue shaded areas are the 95% confidence intervals of the regression lines; the violet areas are the overlaps between the confidence intervals of the sex-specific age-regressions.

Figure 4 shows the evaluation of the subject-specific PQ/RR hysteresis time constants and their comparison with QT/RR hysteresis time constants. These constants were derived from the optimization of the curvilinear regression models. The hysteresis constants derived from the other regression models (not presented) were almost identical and showed the same properties.

While the QT/RR hysteresis constants were marginally albeit significantly shorter in females compared to males (115.7 ± 21.3 s vs 123.7 ± 21.9 s, p = 0.00009), the sex difference of the PQ/RR hysteresis constants was reversed and more substantial (114.9 ± 81.1 s vs 65.4 ± 64.3 s, p < 0.00001). The substantial difference is well visible in the top panels of Fig. 4 that show the cumulative distributions of the hysteresis time constants. The panels of the middle row of Fig. 4 show that the sex differences in the hysteresis time constants were consistent across different study sub-groups defined by the source clinical sites.

Substantial difference between the PQ/RR and QT/RR hysteresis time constants was also found in their age relationship (panels on the bottom row of Fig. 4). While the QT/RR hysteresis constants mildly increased with age (r = 0.175, p = 0.003, +3.69 s per 10 years in females; r = 0.349, p < 0.001, +8.96 s per 10 years in males), the PQ/RR hysteresis constants more steeply decreased with age (r = −0.171, p = 0.004, −13.73 s per 10 years in females; r = −0.213, p < 0.001, −16.04 s per 10 years in males).

The PQ/RR and QT/RR hysteresis time constants were not mutually related (r = −0.025 and r = −0.011 in females and males, both not significant).

Since the P-wave-duration/RR patterns were substantially less steep compared to the PQ/RR and QT/RR patterns (see subsequent sections), the assessment of P-wave-duration/RR hysteresis was more influenced by measurement outliers. We have not found any statistically significant differences between the PQ/RR hysteresis and the P-wave-duration/RR hysteresis. Consequently, the individual PQ/RR hysteresis profiles were also used to study the individual P-wave-duration/RR profiles.

#### Curvatures of the curvilinear regression models

**Figure 5:**
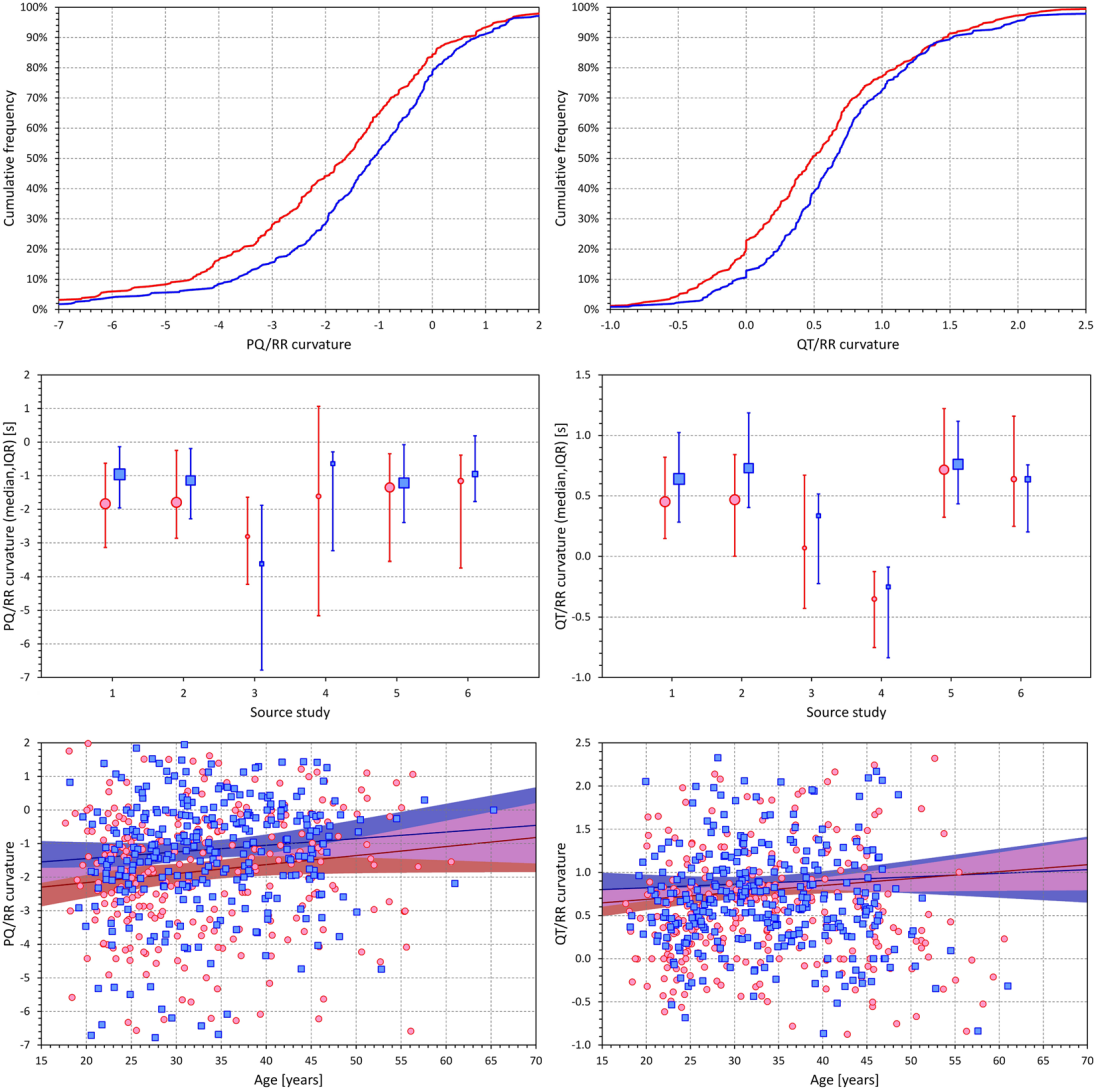
The panels on the top row show the cumulative distribution of subject-specific curvilinear PQ/RR curvatures (the left panel) and of the subject-specific curvilinear QT/RR curvatures (the right panel). The curves of the red and blue lines correspond to the distributions in females and males, respectively. The panels on the middle row show comparisons of the curvatures between females (red circles with red error bars) and males (blue squares with blue error bars) in sub-populations defined by the source clinical centre of the study (see the text for details). The graphs show medians and inter-quartile ranges, the sizes of the marks indicate the numbers of subjects in the sub-populations. The panels of the bottom row show the scatter diagrams of the regression curvatures against the ages of the study subjects. The red/pink circles correspond to females, the blue/azure squares to males. In each panel, the solid red and solid blue lines show the linear regressions between the curvature values and ages in females and males, respectively. The red shaded and blue shaded areas are the 95% confidence intervals of the regression lines; the violet areas are the overlaps between the confidence intervals of the sex-specific age-regressions.

The analysis of the PQ/RR and QT/RR intra-subject curvatures is shown in Fig. 5.

The intra-subject PQ/RR patterns were more curved in females than in males as evident from significantly lower curvature parameters $\gamma$ (−1.90 ± 2.56 in females vs −1.22 ± 2.45 in males, p = 0.001). In both sexes, the PQ/RR patterns were also more curved than the QT/RR patterns ($\gamma$ values 0.54 ± 0.70 in females and 0.73 ± 0.75 in males; p = 0.002 for sex-group comparison; p < 0.00001 for both comparison with PQ/RR curvatures in sex groups). Cumulative distributions of the $\gamma$ values are shown in the panels on the top row of Fig. 5. The panels on the middle row of Fig. 5 show that the sex differences in the curvatures were consistent in different sub-groups by source clinical centre apart from the PQ/RR curvatures in subjects investigated at clinical centre 3 which provided the smallest sub-group (4.7% of study subjects).

The bottom row of panels of Fig. 5 shows that the PQ/RR and QT/RR curvatures tended to decrease (towards higher $\gamma$ values) with advancing age. However, these trends were only marginally significant for PQ/RR curvatures in females (r = 0.119, p = 0.04).

In both sex sub-groups, the PQ/RR and QT/RR curvatures did not correlate with each other.

For reasons of shallow P-wave-duration/RR relationship, the curvatures $\gamma$ of the P-wave-duration/RR patterns showed large spread of values of −1.56 ± 5.61 and −1.28 ± 4.94 in females and males, respectively (no statistical difference between the sex groups). Similar to the PQ/RR patterns, this conformed that the P-wave-duration/RR patterns were substantially more curved than the QT/RR patterns.

#### Slopes of the regression models

**Figure 6:**
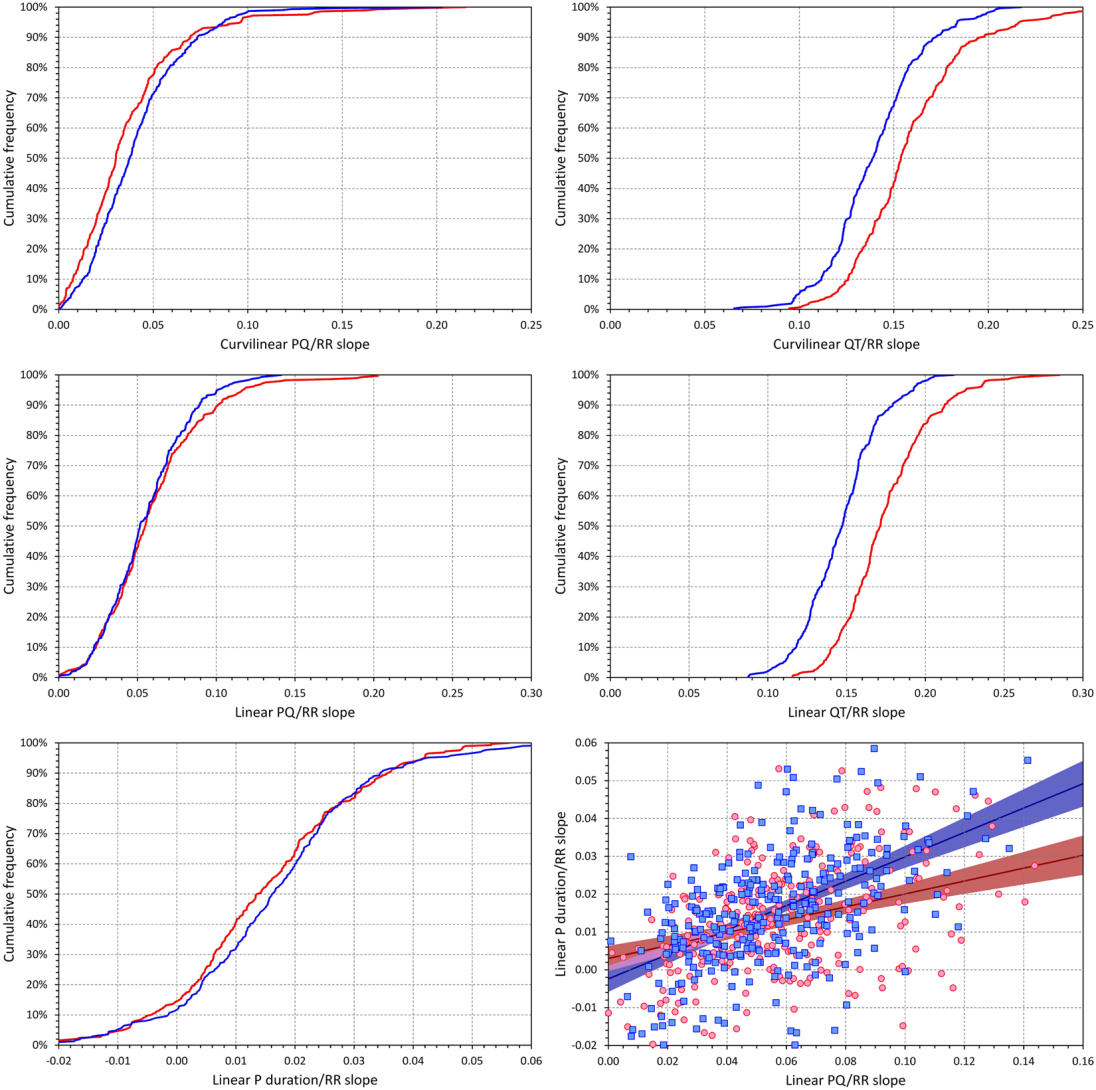
The panels on the top and on the middle row show cumulative distributions of curvilinear and linear regression slopes, respectively. In these two rows, the panels on the right and on the left correspond to PQ/RR and to QT/RR regressions, respectively. The left bottom panel shows the cumulative distribution of the linear slopes of the P-wave-duration/RR relationship (note that the horizontal scale of this panel is different compared to the panels on the two top rows). In all these panels, the curves of red and blue lines correspond to the distributions in females and males, respectively. The bottom right panel shows the scatter diagram between the linear PQ/RR and P-wave-duration/RR slopes. In this panel, the red/pink circles correspond to females, the blue/azure squares to males. The solid red and solid blue lines show the linear regressions between the compared slopes in females and males, respectively. The red shaded and blue shaded areas are the 95% confidence intervals of the regression lines; the violet areas are the overlaps between the confidence intervals of the sex-specific age-regressions.

The top row of panels of Fig. 6 shows the cumulative distributions of PQ/RR and QT/RR curvilinear regression slopes. For PQ/RR regressions, these were very marginally lower in females than in males (0.036 ± 0.032 vs 0.041 ± 0.026, p = 0.047) while the curvilinear QT/RR regressions had substantially steeper slopes in females than in males (0.159 ± 0.032 vs 0.140 ± 0.025, p < 0.00001).

Nevertheless, the curvilinear slopes correspond to the derivative tangent of the curvature at RR of 1 s and are thus influenced by the curvature. Therefore, the middle row of panels of Fig. 6 shows the cumulative distributions of linear PQ/RR and QT/RR slopes. Here, the sex difference in the PQ/RR slopes disappeared (0.060 ± 0.033 vs 0.055 ± 0.026, not significantly different) while the observation of the sex difference of the QT/RR slopes did not change (0.175 ± 0.028 vs 0.147 ± 0.024, p < 0.00001).

As previously observed, PQ intervals were generally less heart rate dependent than the QT intervals. The linear PQ/RR slopes were only 34 ± 19% and 38 ± 19% of the QT/RR slopes in females and males, respectively (p = 0.009 for the sex comparison - the statistical significance was clearly caused by the sex differences in the QT/RR slopes).

The left panel of the bottom row of Fig. 6 shows the cumulative distributions of linear P-wave-duration/RR slopes (note that the horizontal scale of this panel is different since if using the same scale as for the PQ/RR and QT/RR slopes, the figure would not be legible). The slopes in females (0.015 ± 0.015) and in males (0.017 ± 0.016) were not statistically significantly different.

No systematic relationship of the linear PQ/RR slopes to age was found and similarly, the PQ/RR slopes were not systematically related to the QT/RR slopes. Nevertheless, as shown in the right bottom panel of Fig. 6, the PQ/RR and P-wave-duration/RR slopes were significantly correlated (and more so in males than in females).

### Heart rate corrected intervals

**Figure 7:**
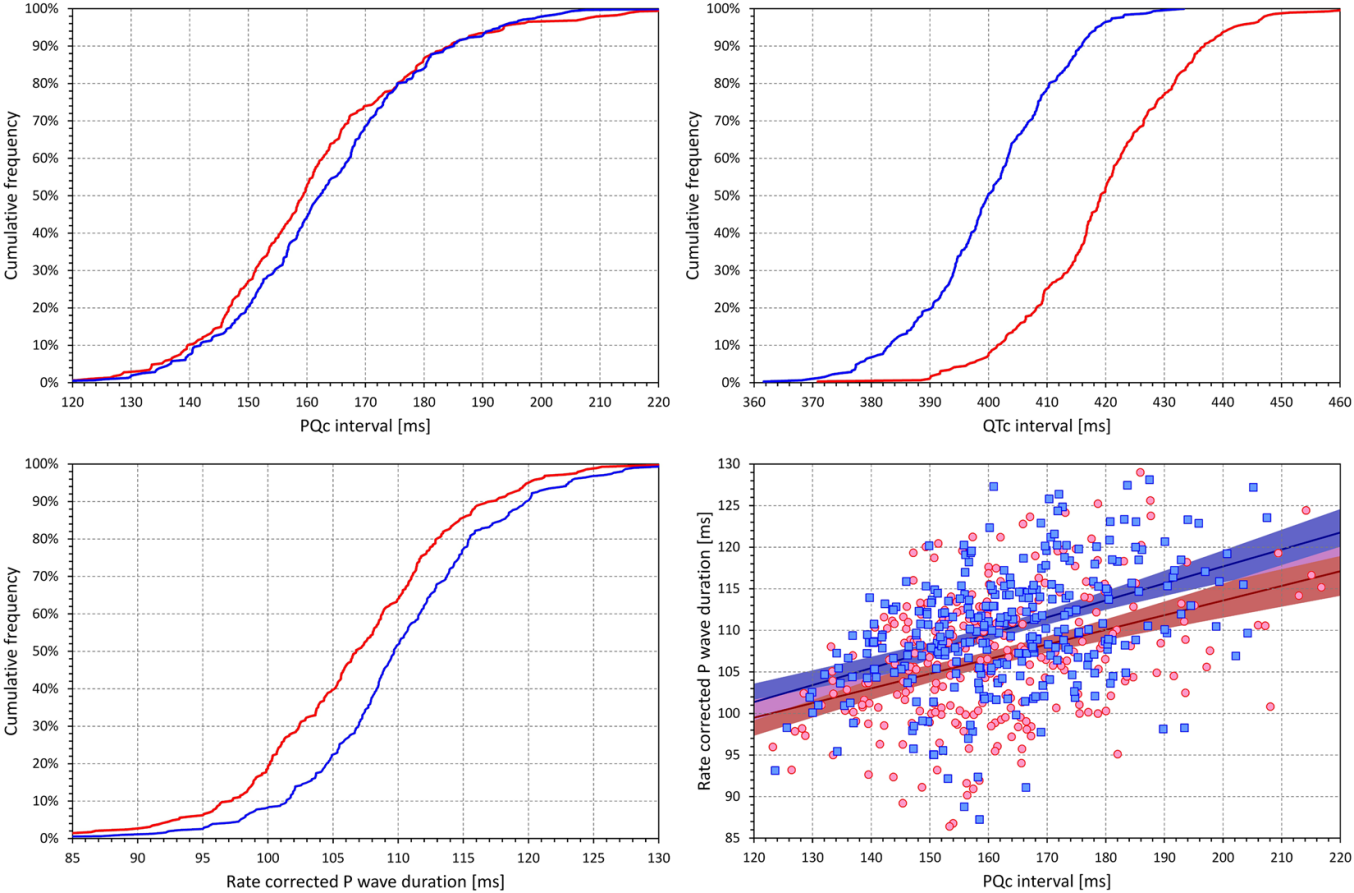
The panels on the top row show the cumulative distribution of individually corrected PQc (left panel) and QTc (right panel) intervals. The left bottom panel shows the cumulative distribution of individually corrected P wave durations. In all these panels, the curves of red and blue lines correspond to the distributions in females and males, respectively. The right bottom panel shows the scatter diagram between the PQc intervals and rate corrected P wave durations. In this panel, the red/pink circles correspond to females, the blue/azure squares to males. The solid red and solid blue lines show the linear regressions between the compared slopes in females and males, respectively. The red shaded and blue shaded areas are the 95% confidence intervals of the regression lines; the violet areas are the overlaps between the confidence intervals of the sex-specific age-regressions.

The cumulative distributions of individually heart rate corrected PQc and QTc intervals (curvilinear models with individually optimized hysteresis corrected RR intervals) are shown in the top row of Fig. 7. As well known, the QTc intervals were significantly longer in females than in males (419.7 ± 13.9 ms vs 400.9 ± 12.4 ms, p < 0.00001). On the contrary, the PQc intervals were not significantly different between the sex groups (161.3 ± 18.6 ms vs 163.3 ± 16.7 ms).

The left bottom panel of Fig. 7 shows the cumulative distributions of rate corrected P wave durations. These were significantly shorter in females compared to males (106.8 ± 8.4 ms vs 110.2 ± 7.9 ms, p < 0.00001). The right bottom panel of Fig. 7 shows that (not surprisingly) the rate corrected P wave durations were significantly correlated to PQc intervals (r = 0.39 and 0.43 in females and males, respectively).

Not surprisingly, the PQc and QTc intervals did not correlate with each other.

### Heart rate bin analyses

**Figure 8:**
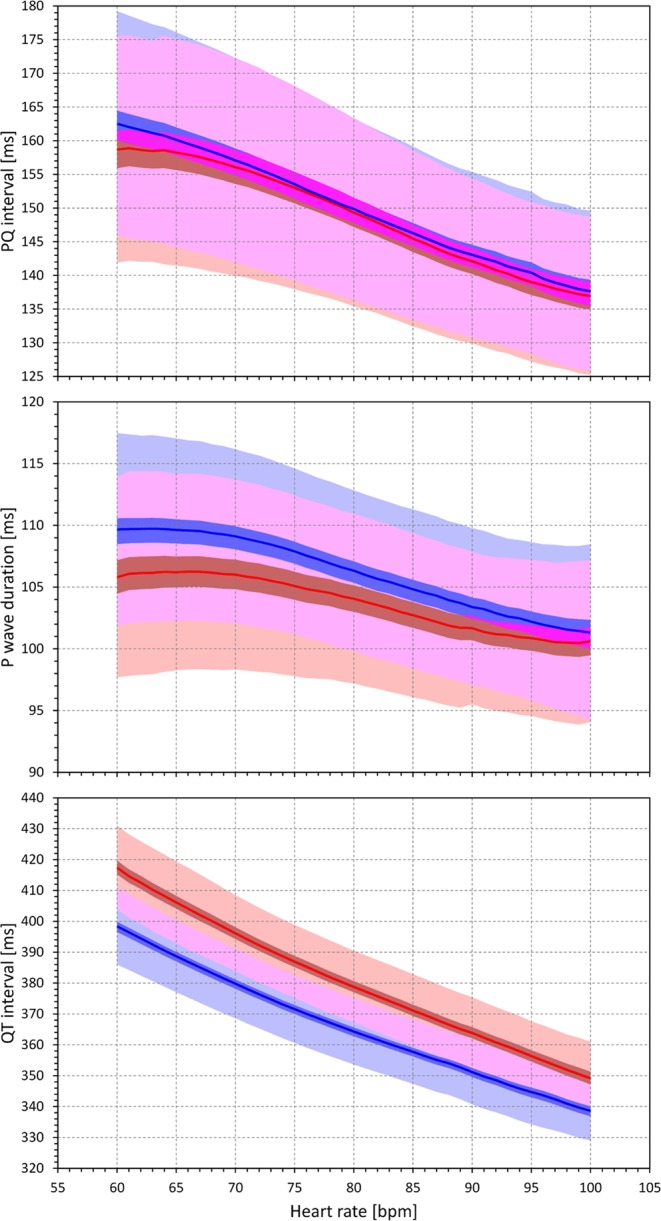
The results of the heart rate bin analysis (see the text for details) of PQ intervals (top panel), P wave durations (middle panel) and QT intervals (bottom panel). The analysis was performed for heart rate bins centred at heart rates between 60 to 100 beats per minute (with bin widths of ±5 beats per minute). The full red and blue lines show the averages of interval measurements per heart rate bins in females and males, respectively. The dark red and blue areas are the 99% confidence bands of the sex-specific averages (the dark violet areas show the overlaps between the confidence bands of both sexes); the light red and light blue areas are the ±standard deviation bands of the sex-specific averages (the light violet areas show the overlaps between standard deviation bands of both sexes).

The heart rate bin analysis of PQ intervals, P wave durations, and QT intervals over the heart rate ranges between 60 and 100 beats per minute is shown in Fig. 8. The Figure confirms the previous observation that the PQ interval durations show little sex differences. The sex difference in the P wave duration is marked at slower heart rates but gradually (and non-linearly) decreases with increasing heart rate. This is reminiscent of the sex difference in the QT interval which, although being reversed, is also decreasing with increasing heart rate.

### Covariates

**Figure 9:**
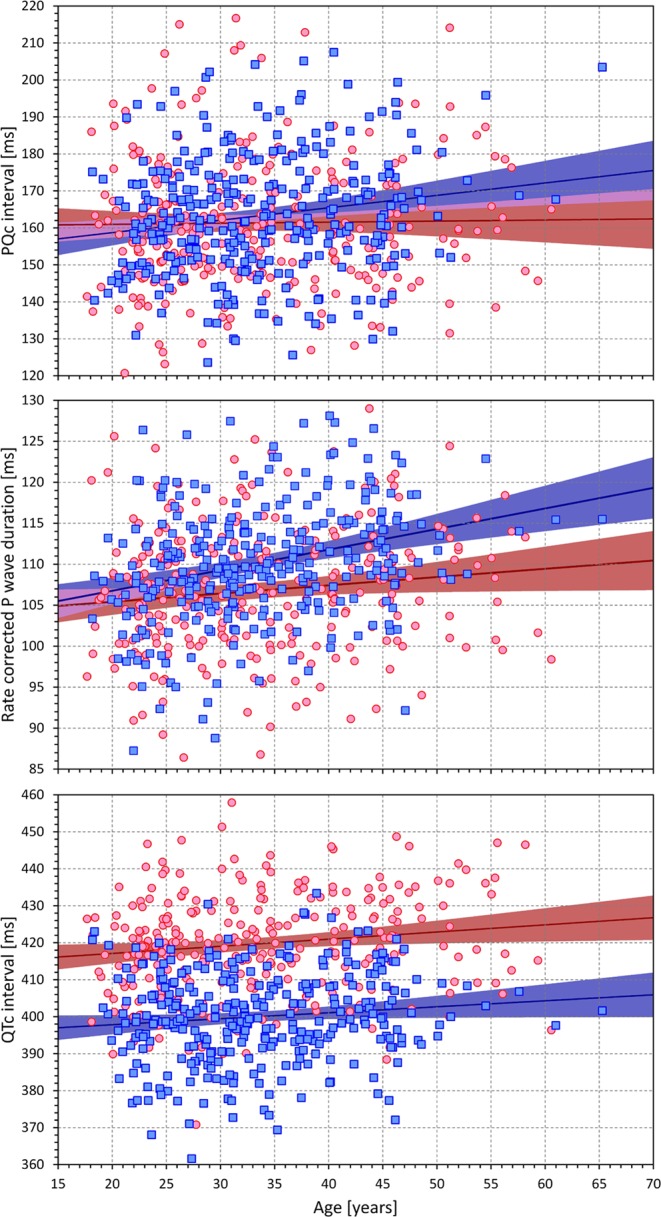
Scatter diagrams of the PQc intervals (top panel), rate corrected P wave durations (middle panel) and QTc intervals (bottom panel) against the ages of the study subjects. The red/pink circles correspond to females, the blue/azure squares to males. In each panel, the solid red and solid blue lines show the linear regressions between the interval durations and ages in females and males, respectively. The red shaded and blue shaded areas are the 95% confidence intervals of the regression lines; the violet areas are the overlaps between the confidence intervals of the sex-specific regressions.

As shown in Fig. 9, QTc intervals were marginally positively correlated with age (r = 0.140, p = 0.017 for females; r = 0.111, p = 0.05 for males). The same marginal increase in PQc with increasing age was observed in males (r = 0.171, p = 0.002) but not in females (r = 0.016, not significant). Similar observations were made with P wave duration (r = 0.121, p = 0.030 for females; r = 0.270, p < 0.0001 for males).

**Figure 10:**
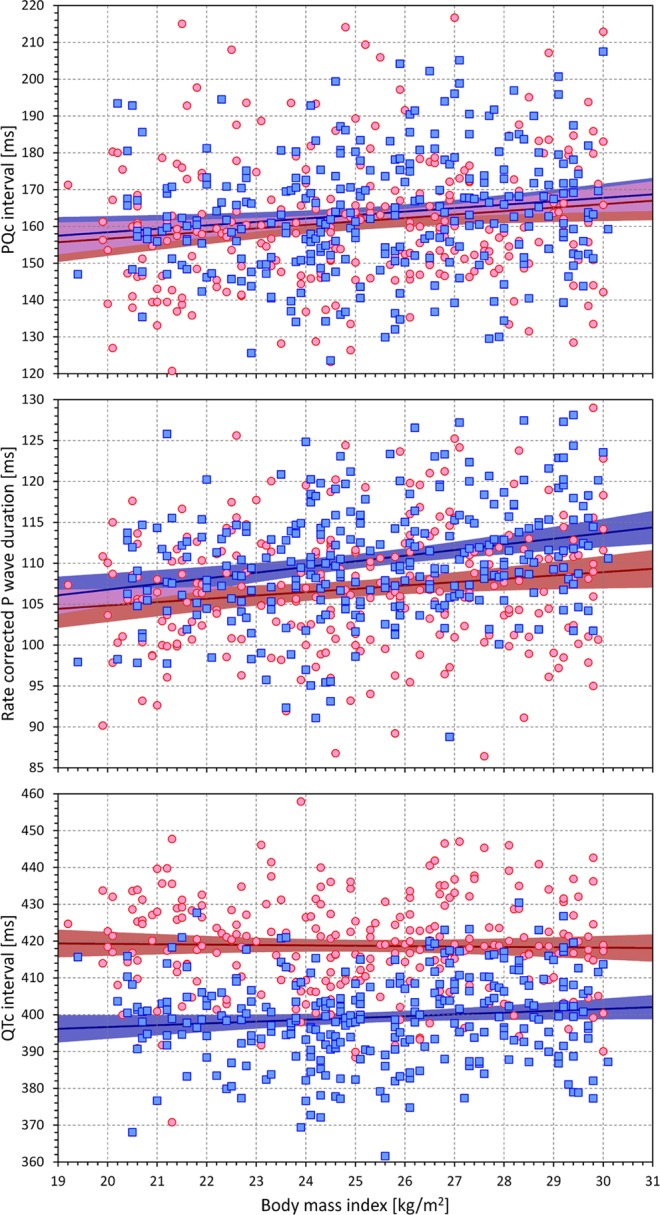
Scatter diagrams of the PQc intervals (top panel), rate corrected P wave durations (middle panel) and QTc intervals (bottom panel) against the body mass index measurements of the study subjects. The layout of the figure and the meaning of the symbols is the same as in Fig. 9.

Figure 10 shows that while QTc intervals were not correlated with body mass index (r = −0.023 and 0.108 for female and males, both not significant), PQc intervals were moderately correlated with body mass index (r = 0.142, p = 0.022 for females; r = 0.150, p = 0.012 for males). Slightly stronger correlations with body mass index were found for the rate corrected P wave durations (r = 0.142, p = 0.022 for females; r = 0.241, p < 0.0001 for males). More importantly, Figure 10 also shows that the sex differences in rate corrected P wave duration were independent of body mass index.

None of the PQ/RR slopes, PQ/RR curvatures, PQ/RR hysteresis constants, P-wave-duration/RR slopes and P-wave-duration/RR curvatures were significantly correlated with body mass index.

## Discussion

The study provides physiologic insight into the PQ interval and P wave duration dynamics. Somewhat more speculatively, the methodology of the study has also potential implications for future clinical applications linking the PQ interval and P wave duration dynamics to outcome and risk of atrial electrophysiologic abnormalities.

Not all observations that we have made are novel. The fact that PQ interval is heart rate dependent has been well known before^16,18^. Soliman and Rautaharju^16^ also proposed that PQ interval is linearly related to heart rate which is consistent with our observation that hyperbolic model fits the data better than a linear model (since the RR interval duration is reciprocal of the heart rate). Other aspects of our observations have not been reported before. The simple fact PQ interval adapts to changing heart rate with a delay is not very remarkable. However, the substantial sex differences in the PQ/RR hysteresis and its relationship to age are perhaps surprising. These observations might be of substantial pathophysiologic importance.

The PQ interval durations reflects combination of intra-atrial and atrioventricular conduction speeds. The duration of the P wave reflects atrial depolarization. The difference between these two intervals (that is, the duration of the isoelectric PQ segment) does not have a clearly defined electrophysiologic meaning since the depolarization signal reaches the atrioventricular node (and thus the atrioventricular conduction is initiated) before the more remote parts of the left atrial myocardium is activated.

The differences in the profiles of the PQ intervals and of P wave durations suggest that both atrial electrophysiology and the atrioventricular nodal conduction are both under autonomic influence. Since the available data do not allow timing the atrioventricular nodal conduction, we can only conclude that autonomic influences at both levels combine and that this combination also exhibits sex differences, probably mediated by hormonal influences^28^.

Speculatively, it seems plausible to propose that by simple closed loop mechanisms, the delay in the response of these conductions speeds to the changing heart rate contributes to the stability of intra-atrial and atrioventricular depolarization. Hence, the substantial sex differences in the PQ/RR hysteresis (with women changing their PQ intervals with a longer delay) together with the differences in PQ/RR slopes and in PQc intervals as well as the marginally shorter P wave durations might all be contributing to the reduced incidence of atrial fibrillation in pre-menopausal women compared to similarly aged men^29,30^. The hypothesis that heart rate changes might decrease intra-atrial depolarization stability seems also consistent with the observation that the risk of atrial fibrillation is increased in younger subjects practicing physical exercise^31^. The decrease of PQ/RR hysteresis time constants with advancing age (as shown in Fig. 4) appears to be in agreement with the increased incidence of atrial fibrillation in older patients^31,32^.

Similar to QT interval hysteresis, the detailed mechanisms of the PQ interval hysteresis are not known. While heart rate changes are well known to be autonomically mediated, the speed of not only parasympathetic (single seconds) but also of sympathetic (20–30 seconds) responses is much faster than the hysteresis delay (in minutes). The same applies to the dynamics of the ion channels involved in cardiac electrophysiologic mechanisms^32^. It is therefore more likely that the mechanisms of hysteresis involve gradual closed loop mechanisms that maintain the equilibrium between the sinus nodal rate and the depolarization and repolarization processes. Clearly, such equilibrium maintenance is bound to be different between depolarization and repolarization processes, as evident from our observations of the QT and PQ hysteresis profiles. Similarly, physiologic mechanisms for the PQ/RR hysteresis responsible for the decline with advancing age are difficult to elucidate. We can only speculate about the influence of autonomic control since this also declines with advancing age.

As already mentioned, sex differences in cardiac electrophysiology are mostly attributed to the influence of sex hormones^28^. Verification of such a concept is clearly beyond the possibilities offered by our data as no hormonal measurements were included in the source clinical studies. In addition to future studies with detailed hormonal measurements, it would also seem appropriate to replicate these analyses in ECG data of children and adolescents^33^ to answer the question of when in life the sex difference appears. In addition, similar to the QT/RR hysteresis which, as shown, exhibits opposite age influence and sex differences, PQ/RR hysteresis might likely be under autonomic control also contributing to both sex and age influences^34^. Having this in mind, novel possibilities of testing atrial electrophysiology and atrial arrhythmia risk might be proposed based on actual measurement of PQ/RR hysteresis during standardized provocative manoeuvres such as tilt-testing or simpler postural provocations that are known to lead to controlled abrupt heart rate changes.

In relation to this, the technology of assessing the PQ/RR hysteresis needs also to be addressed. The distribution curves of the PQ/RR hysteresis time constants cover very broad range of numerical results. While this might be entirely physiologic, it is also possible that the exponential decay model that we used in this investigation is not the optimum technology and that other approaches to the hysteresis assessment might need to be researched^35,36^. Since we used the very same approach to all study subjects, the observed large differences between females and males are undisputable. Nevertheless, the numerical calculations by different hysteresis corrections might lead to different time constants.

The curvatures of the P-wave-duration/RR and PQ/RR relationship might also be contributing to the stability of intra-atrial depolarizations. Visual comparisons of the PQ/RR patterns with QT/RR patterns in Fig. 1 show that the increased curvature means that PQ interval duration is relatively stable until the underlying heart rate increases above a certain (subject-specific) threshold above which the PQ interval progressively shortens. The same appears to apply to the P wave duration. (The population trends shown in Fig. 8 do not fully reflect the individuality of subject-specific curvatures.) Thus, the increased PQ/RR curvatures in females and their marginally lower curvilinear slopes indicated that in females, PQ interval and P wave durations are more stable over a wider range of heart rates. Similar to the sex differences in PQ/RR hysteresis, this might also contribute to the lower incidence of atrial fibrillation in younger females.

Additionally, the sex-specific PQ/RR behaviour might also be of some importance in atrioventricular delay optimization of implantable devices, especially in cardiac resynchronization therapy. Possibly, sex-specific settings (e.g. that of rate adaptive atrioventricular delay) might be beneficial^37^. Nevertheless, compared to healthy individuals, PQ interval behaviour in patients requiring resynchronization therapy is likely influenced not only by the underlying pathology by also by the variety of drugs used in heart failure patients.

As many aspects of these observations appear novel, there is little in the published literature which could be compared to our results apart from the simple findings by Soliman and Rautaharju^16^ that we have already mentioned. Nevertheless, when applying the very same data processing technology to the QT/RR relationship, the results of the analyses were in close agreement with previous independent observations^38,39^ and with existing consensus^40^.

Although the source clinical studies did not provide echocardiographic measurements of heart dimensions, the approximation of body sizes by body mass index showed that none of the observed sex differences were likely related to the heart sizes which are known to be, on average, larger in males compared to females^41^.

### Limitations

Our data and analyses also suffer from several limitations. In addition to the curvilinear regression model, other mathematical forms could be proposed reflecting the shapes of the PQ/RR patterns shown in Fig. 1. We have not performed such investigations. Nevertheless, the regression residuals shown in Table 2 were fairly small and thus the curvilinear model used in the analyses fitted the data reasonably. We have assumed that the subject-specific PQ/RR hysteresis follows the same adaptation profile for both heart rate increases and decreases. This might not be the case. The lack of the separation between heart rate acceleration and deceleration episodes does not impact on the observed sex difference but should still be addressed in the future. Similarly, we have not addressed the possibility of the circadian variation of the PQ/RR profiles. In particular, since the source Holter recordings were obtained solely during day-time hours when the subjects were awake, we were not able to consider day/night differences. The data available for this investigation also did not include P wave morphological characteristics. Finally, all the source data were obtained from healthy subjects for whom we do not have any clinical follow-up. We cannot comment on what the PQ/RR profile might be in cardiac patients, e.g. those with a history of atrial fibrillation ablation.

### Conclusion

Despite these limitations, the study permits to conclude that PQ interval depends on the underlying cardiac cycle length in a highly non-linear fashion and that this non-linearity is more marked in females than in males. Even larger sex difference exists in the speed of the response of the PQ interval duration to heart rate changes which is, on average, much lower in females than in males. P wave duration was found marginally but still significantly shorter in females compared to males. These findings highlight substantial sex differences in atrial and atrioventricular depolarization electrophysiology. It is reasonable to propose that future research of non-invasive risk assessment of atrial arrhythmias should include the characteristics of P wave and PQ interval dynamics.
